# Parallel Farby–Perot Interferometers in an Etched Multicore Fiber for Vector Bending Measurements

**DOI:** 10.3390/mi15121406

**Published:** 2024-11-21

**Authors:** Kang Wang, Wei Ji, Cong Xiong, Caoyuan Wang, Yu Qin, Yichun Shen, Limin Xiao

**Affiliations:** 1 Advanced Fiber Devices and Systems Group, Key Laboratory of Micro and Nano Photonic Structures (MoE), Key Laboratory for Information Science of Electromagnetic Waves (MoE), Shanghai Engineering Research Center of Ultra-Precision Optical Manufacturing, School of Information Science and Technology, Fudan University, Shanghai 200433, China; 21210720014@m.fudan.edu.cn (K.W.); 20210720010@fudan.edu.cn (W.J.); 21110720019@m.fudan.edu.cn (C.X.); 18110720007@fudan.edu.cn (C.W.); 22110720122@m.fudan.edu.cn (Y.Q.); 2 Zhongtian Technology Advanced Materials Co., Ltd., Nantong 226009, China; shenyc@ztt.cn

**Keywords:** Multicore fiber, vector bending sensor, Fabry–Perot interferometer

## Abstract

Vector bending sensors can be utilized to detect the bending curvature and direction, which is essential for various applications such as structural health monitoring, mechanical deformation measurement, and shape sensing. In this work, we demonstrate a temperature-insensitive vector bending sensor via parallel Farby–Perot interferometers (FPIs) fabricated by etching and splicing a multicore fiber (MCF). The parallel FPIs made in this simple and effective way exhibit significant interferometric visibility with a fringe contrast over 20 dB in the reflection spectra, which is 6 dB larger than the previous MCF-based FPIs. And such a device exhibits a curvature sensitivity of 0.207 nm/m^−1^ with strong bending-direction discrimination. The curvature magnitude and orientation angle can be reconstructed through the dip wavelength shifts in two off-diagonal outer-core FPIs. The reconstruction results of nine randomly selected pairs of bending magnitudes and directions show that the average relative error of magnitude is ~4.5%, and the average absolute error of orientation angle is less than 2.0°. Furthermore, the proposed bending sensor is temperature-insensitive, with temperature at a lower sensitivity than 10 pm/°C. The fabrication simplicity, high interferometric visibility, compactness, and temperature insensitivity of the device may accelerate MCF-based FPI applications.

## 1. Introduction

The measurements of bending are crucial in various applications, such as mechanical deformation and structural health monitoring [1,2], medical and industrial robots [3,4,5], and wearable electronics [6,7]. Over the past few decades, the demands for various kinds of bending sensors have increased rapidly [1,8]. Compared to electronic bending sensors [8,9], optical fiber bending sensors have attracted intense interest due to their superiority lightweight, compactness, electromagnetic immunity, and the capability of remote operation.

The fiber-based bending sensors have evolved from a basic one-dimensional curve bending measurement to the more sophisticated spatially two-dimensional bending measurement, commonly known as vector bending sensing, which is able to discriminate the direction in all fiber orientations. MCF inherently possesses advantages in vector bending sensing because the spatial positions of the various cores induce a distinctive strain distribution in the transverse cross-section when the fiber is bent. For instance, a few MCF-based vector bending sensors, such as Bragg gratings (FBGs) [10,11,12,13,14], long period gratings (LPGs) [15,16], Mach–Zehnder interferometers (MZIs) [17,18,19,20,21,22], super-mode interferometers (SMIs) [23,24] and Michelson Interferometers (MIs) [25,26] have been developed. Additionally, the MCF-based Fabry–Perot interferometer (FPI) vector bending sensors have been investigated with great interest due to their compactness and reliability [27,28,29].

A variety of techniques were employed to fabricate the MCF-based FPI sensors, such as resin-connected MCF [29,30,31], the splicing hollow core fiber with MCFs [27,28], and laser micro/nano-manufacturing [32,33]. Ricardo et al. proposed a resin-connected MCF-based FPI curvature sensor by filling the gap between an MCF and a single-mode fiber (SMF) with a transparent photopolymerizable resin, achieving a sensitivity seven times higher than the FBG-based MCF [11]. However, the thermodynamic properties of the polymer resin may weaken the sensing accuracy and reliability in different temperatures. Alternatively, Yang et al. implemented MCF-based FPIs for strain and directional bending measurements, which were fabricated via splicing a fraction of a dual side-hole fiber between a seven-core multicore fiber (MCF) and a multimode fiber [27]. However, it can only discriminate positive and negative directions, not a vector fiber bending sensor. The vector bending sensors can be manufactured by mirror inscriptions [32] and drilling holes in the MCF cores with a femtosecond laser [33]. However, the fabrication instruments and procedures are expensive, which may prevent the wide application of these methods.

In this work, we proposed and demonstrated a simple and effective approach to implement parallel air-cavity FPIs inside an MCF for vector bending sensing, which was fabricated via etching the end-facet of MCF with a hydrofluoric acid solution and splicing two etched MCFs. These seven parallel FPIs were fabricated simultaneously with only one etching and splicing procedure, so the fabrication process was simple and cost-effective. The reflection spectra have a fringe contrast of over 20 dB, which is much higher than the FPIs of similar size fabricated by the femtosecond laser drilling procedure [33]. The curvature sensitivities of three off-diagonal cores have been measured in different fiber orientations, with a maximum sensitivity value of ~0.207 nm/m^−1^, which is approximately four times that of MCF-based FBGs [11]. The reconstruction of the curvature magnitude and bending orientation angle was implemented via the spectral wavelength shifts in two off-diagonal outer-core FPIs. The reconstruction results show that the relative error of magnitude is within 7% with an average of 4.5%, and the absolute error of the orientation angle is within 5°, with an average of 1.8°, comparable to that in [11]. Moreover, the proposed vector bending sensor shows a temperature sensitivity of less than 10 pm/°C, indicating its stability in temperature-perturbed environments. The fabrication simplicity, high interferometric visibility, compactness, and temperature insensitivity of the device may accelerate the MCF-based FPI applications.

## 2. Principle and Fabrication

### 2.1. Principle

**Figure 1:**
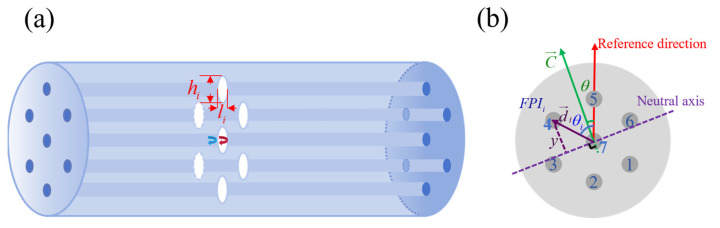
(**a**) Schematic of the proposed vector bending sensor, composed of seven parallel FPIs fabricated inside the MCF. (**b**) The cross-section of the MCF shows the reference direction, bending direction, and directions of the FPIs.

The proposed vector bending sensor is composed of seven parallel air micro-cavities located inside the MCF cores. The schematic of such a device is presented in Figure 1, while the cores and the corresponding FPIs are numbered clockwise from 1 to 7 on the cross-section, as shown in Figure 1b. The air cavity has a spheroidal shape, and the polar and equator diameters are *l_i_* and *h_i_*, respectively. As shown in Figure 1a, in the corresponding cavity, multiple reflections can occur when the light is transmitted through each core, and the two main coherent reflected lights with a phase difference can meet in each core, resulting in interference resonance. The peaks of the spectrum correspond to constructive interference, while the valleys of the spectrum represent destructive interference, where the reflected waves cancel each other out.

Considering the intensities of the reflected beams as *I*_1_ = *I*_0_*R* and *I*_2_ = *I*_0_(1 − *R*)^2^*Rη*, here, *I_0_* is the intensity of the initial beam, and *R* is the reflection coefficients for the Fresnel reflection on the interface between the fiber core and the air. And *η* is the incidence rate of reflected light [34]. The intensity resultant resulting from the interference can be represented as follows [29]:

(1) $$I=I_{1}+I_{2}+2\sqrt{I_{1}I_{2}}\cos(\phi_{1}-\phi_{2})$$

When the rear concave surface has a proper curvature radius, the reflected beam from the rear surface will be reflected back to the core of the fiber by the concave mirror focusing principle [35]. So, the device will exhibit a larger fringe contrast than the FPs composed of planar surfaces.

The phase difference between the two beams and can be expressed as follows:

(2) $$\phi_{1}-\phi_{2}=\frac{2\pi }{\lambda }2nl$$

where *λ* is the free-space wavelength, and *n* and *l* are the refractive index (RI) and the length of the FP cavity, respectively. Therefore, the changes in RI and length of the cavity can affect the optical path difference between the coherent beams, resulting in variations in the constructive and destructive interference wavelength, which is represented in the spectrum through the shifts in peaks and valleys.

**Figure 2:**
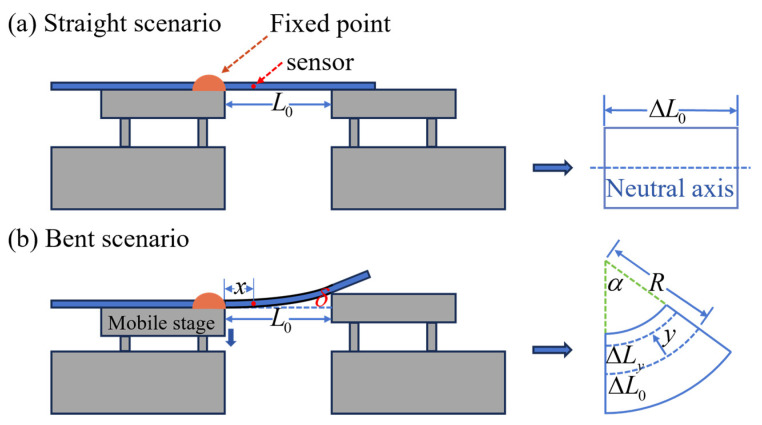
The principle of bending measurement. (**a**) Optical fiber is in a straight state. (**b**) The fiber is bent when the left terminal moves down a distance *δ* from its original position. The images on the right are the transversal section of a small section of fiber (∆*L*_0_), respectively, in a straight and bent scenario.

Once the fiber is bent, each FPI inside the MCF is compressed or stretched to varying degrees, causing different changes in RI and the length of each FP cavity. The principle of the bending measurement is shown in Figure 2. Initially, one end of the fiber is fixed to the left stage, and the other end is free. Adjust the height of the right stage to be the same as that of the left stage; in this case, the MCF remains straight, with a certain distance $L_{0}$ between the two stages [Figure 2a]. When the left stage drives the left fixed point down a distance (defined as deflection *δ*), the fiber bends, as depicted in Figure 2b. The relationship between the deflection and the curvature of the fiber can be described as [36]:

(3) $$\delta =\frac{1}{R}=\frac{C\left(x\right)L_{0}^{3}}{3\left|\left(x-L_{0}\right)\right|}$$

where R is the radius of the curvature and *C*(*x*) is the curvature of the fiber at the horizontal distance *x* from the fixed point. Therefore, the curvature of the fiber is variable depending on the fiber location, but it can be regarded as a constant for a small section, as shown in the right images of Figure 2b.

The bending of FPI is caused by the internal longitudinal strain (*ε*), along the fiber, which is defined as follows:

(4) $$\varepsilon =\frac{\Delta l}{l_{0}}$$

where *l*_0_ is the initial length, ∆*L*_0_ is the change in length. According to the geometric relationship shown in the right images of Figure 2b, *ε* can be expressed as follows:

(5) $$\varepsilon =\frac{\Delta L_{y}-\Delta L_{0}}{\Delta L_{0}}=-\frac{y}{R}=-Cy$$

where ∆*L*_0_ is the optical fiber arc length at the neutral axis and ∆*L_y_* is the arc length at the radial distance y from the neutral axis. The device fabricated inside of the fiber is located as *x* away from the fixed point. The strain of FPI_7_ is zero, because it is located at the neutral axis. The strain of the outer core can be written as follows:

(6) $$\varepsilon_{i}=-Cy=-\vec{C}\cdot {\vec{d}}_{i}=-Cd\cos(\theta -\theta_{i})$$

where the vector $\vec{d}$ has a magnitude of the core pitch *d*, with the direction of the central core towards the external cores, as is shown in Figure 1b. The direction of the central core towards core5 is selected as the reference direction; the *θ* refers to the angle between the direction of the bending and the reference direction; and each core is positioned *θ_i_* from the reference direction, as is depicted in Figure 1b. When the difference between the azimuth angle of FPIs and the curvature orientation angle $\left(\theta -\theta_{i}\right)$ is at angles ranging from 90 to 270 degrees, *ε_i_* is positive and the FPI*_i_* is stretched. On the contrary, *ε_i_* is negative, and FPI*_i_* is compressed.

It is possible to express the wavelength shift caused by the strain of an *m*th dip wavelength of the FPI*_i_*, such as the following:

(7) $$\Delta \lambda_{i}=\lambda_{m}\left(1+\frac{1}{n}\frac{\partial n}{\partial \varepsilon_{i}}\right)\varepsilon_{i}$$

where the first term, *λ_m_ε_i_*, corresponds to the change in cavity length, and the second term $\lambda_{m}\frac{1}{n}\frac{\partial n}{\partial \varepsilon_{i}}\varepsilon_{i}$ corresponds to the RI change in the cavity due to the elastic–optical effect. Since the elastic–optic coefficient of air can be negligible [37], Equation (7) can be derived as follows:

(8) $$\Delta \lambda_{i}=-\lambda_{m}Cd\cos(\theta -\theta_{i})$$

Any two functions of off-diagonal outer cores, such as core2 and core4, can form a matrix. Therefore, the bending orientation angle *θ* and curvature magnitude *C* can be acquired with the matrix.

**Figure 3:**
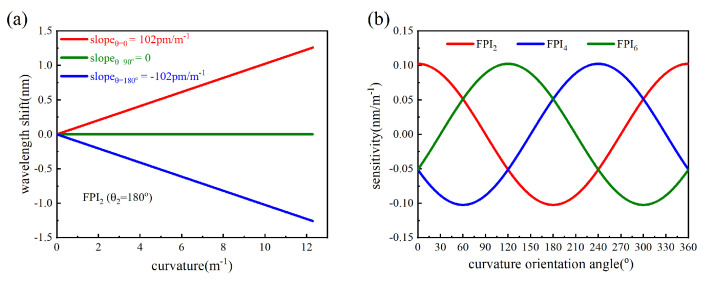
(**a**) The wavelength shifts of FPI_2_ as functions of curvature when *θ* = 0°, *θ* = 90° and *θ* = 180°, respectively. (**b**) The curvature sensitivities of FPI_2_, FPI_4_, and FPI_6_ as functions of the fiber orientation in cartesian coordinates.

In order to illustrate the relationship between the wavelength shift and curvature, as well as the relationship between bending sensitivity and bending direction more clearly, we simulated Equation (8) using MATLAB 2022a, as shown in Figure 3. The wavelength shifts in FPI_2_ showed a linear relationship with curvature when *θ* = 0°, *θ* = 90° and *θ* = 180°, with sensitivities of 102 pm/m^−1^, 0, and −102 pm/m^−1^, respectively. And the bending sensitivities of FPI_2_, FPI_4_, and FPI_6_ exhibit a sinusoidal relationship with the curvature orientation angle.

### 2.2. Fabrication

**Figure 4:**
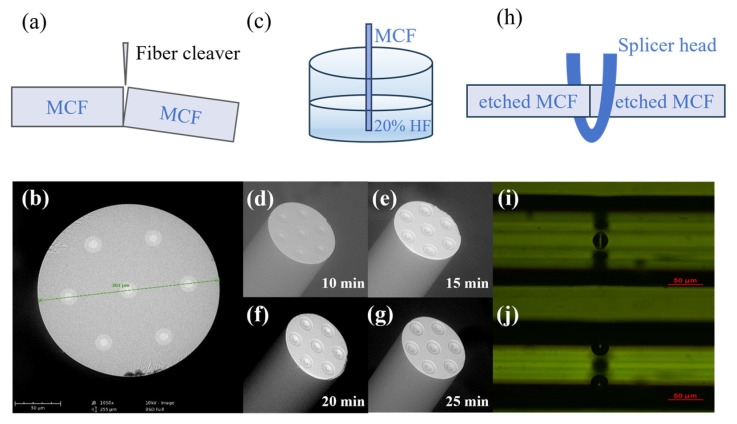
The device fabrication process. (**a**) A piece of MCF was cut to obtain the flat fiber end facet. (**b**) The cross-section of the MCF. (**c**) The flat cleaved fiber tip was exposed to 20% hydrofluoric acid (HF) at 25 °C. (**d**–**g**) The etched facets over different etching times. (**h**) Two sections of MCFs with etched facets were spliced together. (**i**,**j**) The spliced joint focused on the central core and side cores.

The fabrication process of parallel air cavity FPIs in the MCF is shown in Figure 4. Firstly, a piece of MCF was cut to obtain a flat fiber end facet by a fiber cleaver (Fujikura CT-104, Fujikura Ltd., Tokyo, Japan) in Figure 4a. The MCF is composed of seven cores, one at the center and six others arranged in a hexagonal shape. The fiber has an outer diameter of 201 μm, a core diameter of about 8.2 μm, and a core pitch of 66 μm, as depicted in Figure 4b. Next, the flat cleaved fiber tip was exposed to 20% hydrofluoric acid (HF) at 25 °C, as shown in Figure 4c. As the silica doped with GeO_2_ was etched at a higher rate than the pure silica when exposed to HF [38], seven air holes could be formed at seven cores simultaneously. Figure 4d–g show the etched facets over different etching durations between 10 min and 25 min. The depth of the air holes is deeper with the extension of etching time. It is interesting to note that the ring pattern on the air hole surface is formed after etching, which is due to the doped pattern of MCF. Finally, two MCFs with an etching duration of 25 min were spliced together by a fusion splicer (Vytran GPX-3400, Vytran LLC, Paramus, NJ, USA), as shown in Figure 4h. Due to the melting process during the fusion splicing, seven micro-holes rapidly expanded into elliptically spherical air cavities, as shown in Figure 4i,j. The images show a center cavity with a polar diameter of 24.18 μm and off-center cavities with a polar diameter of 26.05 μm, respectively, with an equator diameter of 36 μm for both the center and off-center cavities. The relatively small difference in polar diameters between the center cavity and the off-center cavities may be attributed to the difference in the fusion temperature distribution along the fiber radial direction.

## 3. Experiment Results and Discussion

### 3.1. Characterization

**Figure 5:**
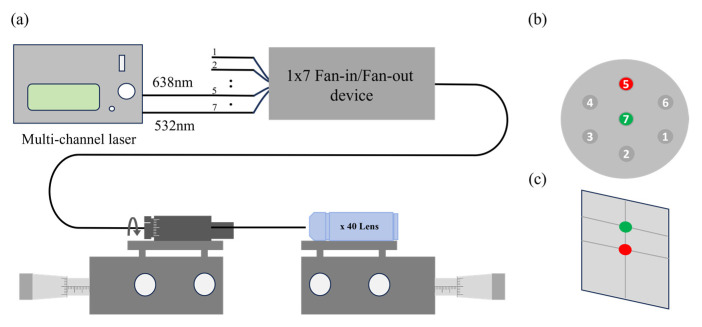
The alignment system. (**a**) The Setup. (**b**) A reference fiber angular position. (**c**) The red-light spot is just below the green-light spot.

Prior to spectral measurement, the MCF bending experiment device was calibrated to ensure the continuity and accuracy of the measurement. The sample was spliced with an MCF fan-in/out device fabricated in [39], and the pigtail of the MCF was cut at an angle of 8° to eliminate the reflection of the fiber end facet. The alignment system is shown in Figure 5a. Two laser beams of green light (532 nm) and red light (638 nm) emitted from the multi-channel laser were connected to core5 and core7. The fiber terminal was held on the fiber rotator (Thorlabs HFR007, Thorlabs, Inc., Newton, NJ, USA) that fixed on a 3D translation stage. The optical fiber end facet was imaged by a 40 X objective lens in front of the fiber tip placed on another 3D translation stage, and the red and green spots appeared on a screen. The reference direction was determined to be straight up by rotating the fiber rotator, as shown in Figure 5b. When the red-light spot is just below the green-light spot in the process of rotating the optical fiber rotator, it can be proved that core5 is just above core7 on the fiber end facet; that is, the reference direction is straight up [Figure 5c].

**Figure 6:**
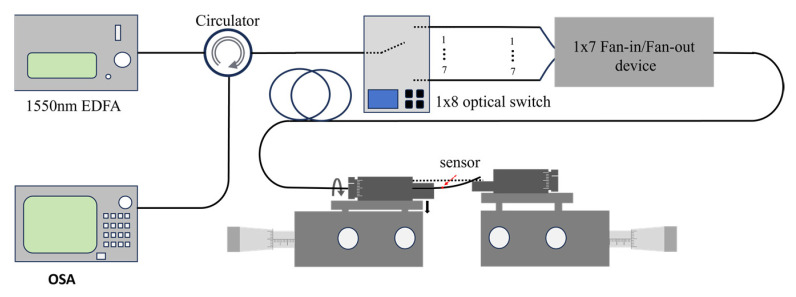
Schematic of interrogation system.

After the alignment process, the alignment system was replaced by the interrogation system, which included a broadband EDFA light source, an optical spectrum analyzer, and an optical switch (1 × 8) for the measurement of the reflection spectrum, as shown in Figure 6.

**Figure 7:**
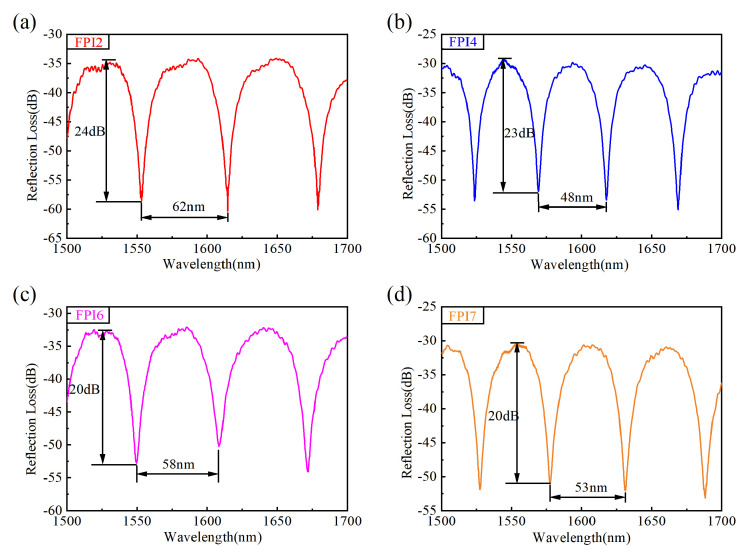
The reflection spectra of FPI_2_, FPI_4_, FPI_6_, and FPI_7_.

Figure 7 plots the reflection spectra of the central cavity (FPI_7_) and the selected outer cavities (FPI_2_, FPI_4_, and FPI_6_) when the MCF is straight. The reflection spectra of all spheroidal air cavities have a fringe contrast over 20 dB, which is 12 dB larger than those of cavities with a similar polar diameter and equator diameter fabricated by the femtosecond laser drilling method [33].

### 3.2. Vector Bending Measurement

The vector bending characteristics of the sensor were implemented using the interrogation system shown in Figure 6. The sensor is 2 mm away from the fixed left point, and the distance is 24 mm between the two stages. The curvature magnitude can be changed by adjusting the height of the left displacement stage. Then, the curvature direction can be changed by rotating the fiber rotator. To simplify experimental operation and data processing, the vector bending of three non-diagonal cores (core2, core4, core6) was measured to obtain curvature sensitivity and directional reconstruction. The measured curvature ranges from 0 to 12.3 m^−1^, with a step of 2.05 m^−1^. The measured direction angle ranges from 0° to 360° with a step of 30°.

**Figure 8:**
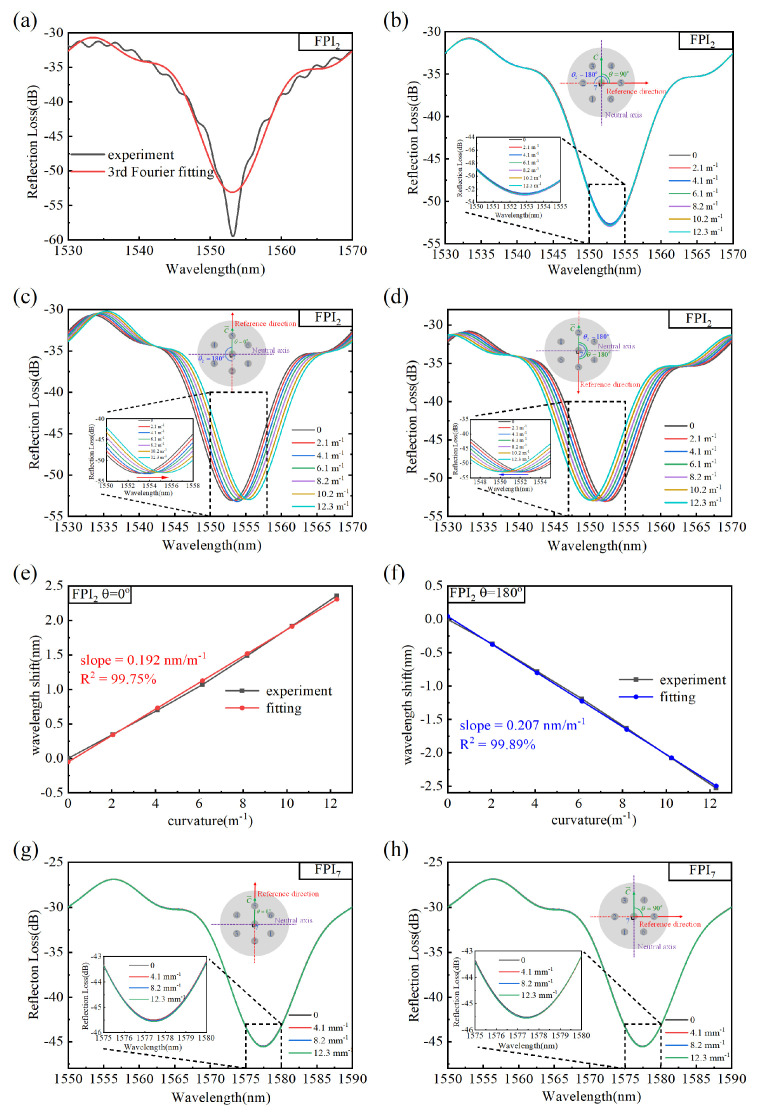
(**a**) The reflection spectrum of FPI_2_ and the third-order Fourier fitting curve. (**b**–**d**) The reflection spectra of FPI_2_ at different curvatures in fiber orientations *θ* = 90°, *θ* = 0° and *θ* = 180°, respectively. (**e**,**f**) The dip wavelength as functions of curvature when *θ* = 0° and *θ* = 180°, respectively. (**g**,**h**) The reflection spectra of FPI_7_ at different curvatures in fiber orientations *θ* = 0° and *θ* = 90°, respectively. The bending characteristics of the central cavity (FPI_7_) in different fiber orientations were also investigated. (**g**,**h**) show the evolution of the reflection spectra of the central cavity with curvature changes in two orthogonal fiber orientations. Since the FPI_7_ is on the neutral plane, the spectrum does not undergo the wavelength shift with the curvature change, regardless of whether θ is 0° or 90°.

The results of core2 in vector bending measurement are used as examples (similar to the results of core4 and core6) to demonstrate the performance of the MCF bending sensor. As shown in Figure 8a, the original spectrum (the black line) has a dip at 1553 nm. However, the high-frequency fluctuations superimposed on the spectrum cause the interference spectrum to deviate from the ideal pattern; as such, it may be difficult to find the true dip. The Fourier series fitting method has proven to be effective in terms of addressing the resonant wavelength shift problem in [40]. Therefore, we remove the high-frequency disturbance of the original spectra by the Fourier fitting, as the red curve shown in Figure 8a suggests. The evolution of reflection spectra with curvature variation is shown in Figure 8b (fiber orientations *θ* = 90°), Figure 8c (*θ* = 0°), and Figure 8d (*θ* = 180°), respectively. The insets are the local spectra near the dip. As the curvature magnitude increases, the spectra are invariant (fiber orientations *θ* = 90°), red-shifted (*θ* = 0°), and blue-shifted (*θ* = 180°), respectively. The azimuth angle of FPI_2_ (*θ*_2_) was set to 180° after the alignment process; thus, the shift direction of the spectrum measured in the experiment with different fiber orientations conforms to Equation (8). The invariant, red-shifted, and blue-shifted spectra indicate that the cavity is unstressed, stretched, and compressed, respectively. The highest positive sensitivity and negative sensitivity can be obtained at *θ* = 0° and *θ* = 180°, respectively. Figure 8e,f show the dip wavelength as a function of curvature, and the positive sensitivity is 0.192 nm/m^−1^, and the negative sensitivity is −0.207 nm/m^−1^, respectively, which is about four times that of MCF-based FBGs [11]. The values are larger than the value of 0.102 nm/m^−1^ calculated by Equation (8), which can be attributed to the fact that the actual curvature is larger than that calculated by Equation (3) due to the existence of air cavities affecting the uniformity of the glass fiber.

**Figure 9:**
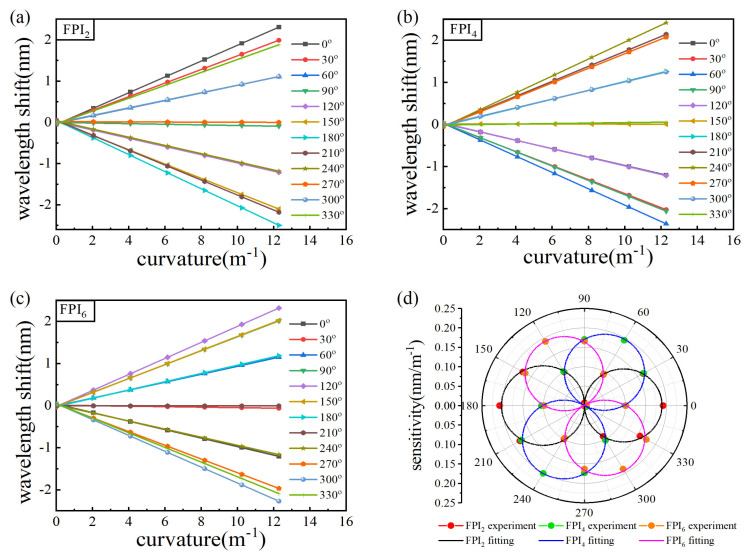
(**a**–**c**) The dip wavelength of FPI_2_, FPI_4_, and FPI_6_ as functions of curvature in all fiber orientation angles. (**d**) The curvature sensitivities of the three FPIs as the function of the fiber orientation in polar coordinates.

The dip wavelength of FPI_2_, FPI_4_, and FPI_6_ as functions of curvature in all fiber orientation angles is shown in Figure 9a–c, respectively. The dip wavelength of FPIs varies linearly with curvature in different fiber orientations. While the slope of the fitted line is different with the change in fiber orientations, indicating that the curvature sensitivity of FP cavity is dependent on the bending direction, the maximum positive sensitivities of FPI_2_, FPI_4_ and FPI_6_ are 0.192 nm/m^−1^, 0.201 nm/m^−1^, 0.190 nm/m^−1^, and the maximum negative sensitivities are 0.207 nm/m^−1^, 0.194 nm/m^−1^, and 0.189 nm/m^−1^, respectively. The values are approximately similar, which verifies that the FPIs in different cores fabricated by our method have good repeatability. In order to visualize the bending-direction dependence clearly, we represent the curvature sensitivities of the three FPIs as a function of the fiber orientation angles in polar coordinates, as is shown in Figure 9d.

The angular dependence of the bend sensitivities for the three outer core FPI cavities show an elegant “8”-shaped pattern, showing good symmetry. Additionally, the curvature sensitivities of FPI_2_, FPI_4_, and FPI_6_ reach the maximum when *θ* = 0°, *θ* = 240°and *θ* = 120°respectively, which verifies that the above alignment process is reliable.

**Figure 10:**
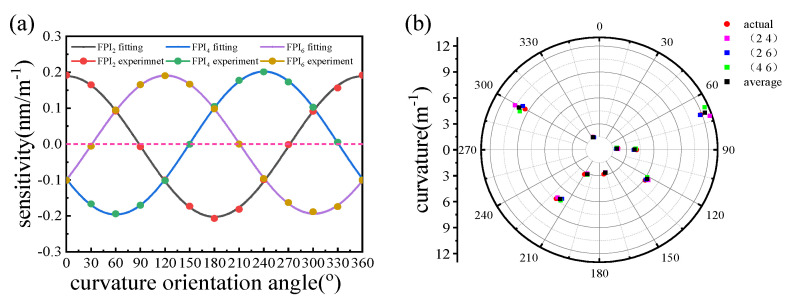
(**a**) The curvature sensitivities of FPI_2_, FPI_4_, and FPI_6_ as functions of the fiber orientation in cartesian coordinates. (**b**) The actual and reconstructed values demodulated by different combinations of the three FPIs for nine random curvature magnitudes and orientations in polar coordinates.

In order to obtain the specific sensitivity expression of every FPI, we represent the curvature sensitivities of the three FPIs as functions of the fiber orientation in cartesian coordinates, as shown in Figure 10a. The expression of the sinusoidal fitting curve can be written as follows:

(9) $$S_{2}=(-0.006524+0.1957\cos(0.0175\theta )+0.002104\sin(0.0175\theta )){\mathrm{nm}/m}^{-1}$$

(10) $$S_{4}=(-0.002587-0.09925\cos(0.0175\theta )-0.1726\sin(0.0175\theta )){\mathrm{nm}/m}^{-1}$$

(11) $$S_{6}=(-0.001448-0.09804\cos(0.0175\theta )+0.1655\sin(0.0175\theta )){\mathrm{nm}/m}^{-1}$$

where $S_{2}$, $S_{4}$, $S_{6}$ are the sensitivities of the three FPIs, respectively.

In order to test the performance of the system, we measured a set of spectra for nine random different curvature magnitudes $C_{1\sim 9}$ in the range of 0 to 12.3 $m^{-1}$ and orientations angles $\theta_{1\sim 9}^{R}$ from 0° to 360°. For a set of curvature magnitudes $C_{1}$ and orientation angles $\theta_{1}^{R}$, the wavelength shifts in the three cores are $\Delta \lambda_{1}^{2}$, $\Delta \lambda_{1}^{4}$, $\Delta \lambda_{1}^{6}$. The relationship between the wavelength shift and the curvature magnitude can be expressed as follows:

(12) $$\Delta \lambda_{1}^{2}=C_{1}(-0.006524+0.1957\cos(0.0175\theta_{1}^{R})+0.002104\sin(0.0175\theta_{1}^{R}))$$

(13) $$\Delta \lambda_{1}^{4}=C_{1}(-0.002587-0.09925\cos(0.0175\theta_{1}^{R})-0.1726\sin(0.0175\theta_{1}^{R}))$$

(14) $$\Delta \lambda_{1}^{6}=C_{1}(-0.001448-0.09804\cos(0.0175\theta_{1}^{R})+0.1655\sin(0.0175\theta_{1}^{R}))$$

Any combination of two of these equations can solve $C_{1}$ and $\theta_{1}^{R}$. Thus, three combinations (FPI_2_ and FPI_4_, FPI_2_ and FPI_6_, FPI_4_ and FPI_6_) can be used to solve the parameters, and the final value can be averaged from the three reconstructions, giving a more accurate value [11]. The nine reconstructed values (C1~9Re, θ1~9Re) through different combinations and the actual values ($C_{1\sim 9}$, $\theta_{1\sim 9}^{R}$) match well with each other in Figure 10b.

**Figure 11:**
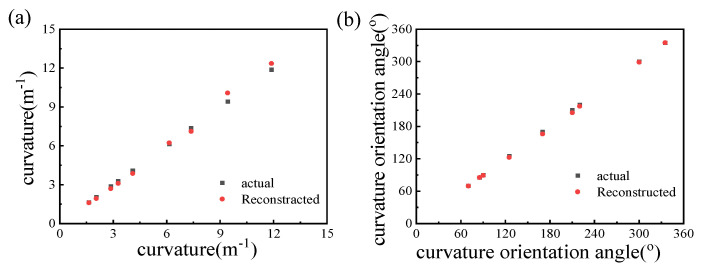
The reconstructed and actual (**a**) curvature magnitudes and (**b**) orientation angles.

The nine reconstructed average results are compared with the actual data in cartesian coordinates to calculate the error, as shown in Figure 11.

The relative errors of the curvature magnitude are within 7%, with an average of 4.5%. The absolute errors of the orientation angles are within 5°, with the average error at 1.8°. The results showed high accuracy and good reliability of the method of reconstructing the vector curvature in a wide range.

### 3.3. Temperature Experiment

**Figure 12:**
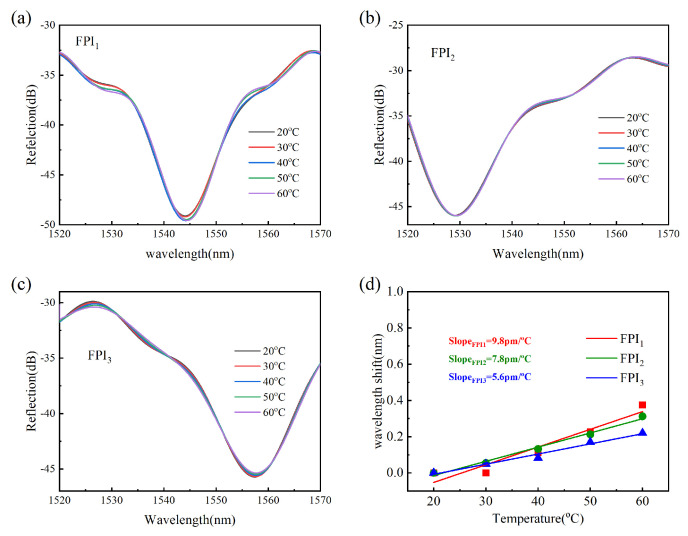
(**a**–**c**) The reflection spectra of FPI_1_, FPI_2_, and FPI_3_ at different temperatures. (**d**) Wavelength shift as a function of temperature for three FPIs.

The temperature characteristic of the proposed sensor was investigated. In the experiment, the sensor was put into a heating oven. The temperature gradually increased from 20 °C to 60 °C with a step of 10 °C. The sensing spectra of three FPIs numbered FPI_1_, FPI_2_, and FPI_3_ presented wavelength red-shift, and the sensitivities were 9.8 pm/°C, 7.8 pm/°C and 5.6 pm/°C, respectively, as shown in Figure 12d. The small difference may be caused by variations in the shape and size of the cavity. Therefore, the proposed device has temperature insensitivity, which can be attributed to the fact that the effective RI of air in the cavity hardly changes with temperature perturbation.

We also compared our results with the previously reported works in Table 1, the parallel FPIs in an MCF fabricated in this way have fringe contrast over 20 dB in the reflection spectra, which is 6 dB higher than the previously reported works.

## 4. Conclusions

In summary, we have proposed and demonstrated experimentally a vector bending sensor based on seven parallel air FPI cavities fabricated by hydrofluoric acid etching and splicing in an MCF. The FPIs exhibited high fringe contrast over 20 dB in the reflection spectra, which is 12 dB higher than previous results manufactured by a femtosecond laser. Such a device exhibits a maximum vector curvature sensitivity of 0.207 nm/m^−1^ in a wide curvature range from 0 to 12.3 m^−1^. The curvature sensitivity of parallel FPI cavities is characterized in detail. The reconstruction of nine pairs of bending magnitudes and orientation angles shows that the average relative error of curvature magnitude is 4.5% and the average absolute error of orientation angle is 1.8°, proving the reliability of the sensor. Moreover, the sensor exhibits pm-level temperature sensitivity. This simple and compact vector bending sensor may accelerate the MCF-based FPI applications.

## Figures and Tables

**Table 1:** Characteristics of the parallel FPIs in multicore fiber.

Methods of Fabrication	The Size of the Device (μm)	Fringe Contrast (dB)	The Maximum Bending Sensitivity (pm/m^−1^)	The Temperature Sensitivity (pm/°C)	Ref.
Cold splicing	600	18	420	176 (need temperature compensation)	[29]
Dual side-hole fiber splicing	135	0.9	242.5	4.1	[27]
Silica capillary optical fiber splicing	225	13	200.6	0.67	[28]
Reflection mirrors fabricated by femtosecond laser	1000	13.5	48.03 nm/mm equivalent to 2.9 nm/m^−1^	532 (need temperature compensation)	[32]
Femtosecond laser micro-holes drilling and splicing	26	8.7	-	0.74	[33]
Corrosion and splicing	26.05	24	200.6	9.8	This work

## Data Availability

The original contributions presented in the study are included in the article, further inquiries can be directed to the corresponding author.

## References

[1] Wang Q., Liu Y. (2018). Review of optical fiber bending/curvature sensor. Measurement.

[2] Ma J.J., Asundi A. (2001). Structural health monitoring using a fiber optic polarimetric sensor and a fiber optic curvature sensor-static and dynamic test. Smart Mater. Struct..

[3] Amanzadeh M., Aminossadati S.M., Kizil M.S., Rakic A.D. (2018). Recent developments in fibre optic shape sensing. Measurement.

[4] Bai H.D., Li S., Barreiros J., Tu Y.Q., Pollock C.R., Shepherd R.F. (2020). Stretchable distributed fiber-optic sensors. Science.

[5] Meerbeek I.M.V., Sa C.M.D., Shepherd R.F. (2018). Soft optoelectronic sensory foams with proprioception. Sci. Robot.

[6] Dong K., Peng X., Wang Z.L. (2020). Fiber/Fabric-Based Piezoelectric and Triboelectric Nanogenerators for Flexible/Stretchable and Wearable Electronics and Artificial Intelligence. Adv. Mater..

[7] Xiang L., Zeng X.W., Xia F., Jin W.L., Liu Y.D., Hu Y.F. (2020). Recent Advances in Flexible and Stretchable Sensing Systems: From the Perspective of System Integration. ACS Nano.

[8] Saggio G., Orengo G. (2018). Flex sensor characterization against shape and curvature changes. Sens. Actuators A-Phys..

[9] Lee B.G., Lee S.M. (2018). Smart Wearable Hand Device for Sign Language Interpretation System with Sensors Fusion. IEEE Sens. J..

[10] Barrera D., Madrigal J., Sales S. (2017). Tilted fiber Bragg gratings in multicore optical fibers for optical sensing. Opt. Lett..

[11] Hou M.X., Yang K.M., He J., Xu X.Z., Ju S., Guo K.K., Wang Y.P. (2018). Two-dimensional vector bending sensor based on seven-core fiber Bragg gratings. Opt. Express.

[12] Bao W.J., Sahoo N., Sun Z.Y., Wang C.L., Liu S., Wang Y.P., Zhang L. (2020). Selective fiber Bragg grating inscription in four-core fiber for two-dimension vector bending sensing. Opt. Express.

[13] Feng D.Y., Zhou W.J., Qiao X.G., Albert J. (2015). Compact Optical Fiber 3D Shape Sensor Based on a Pair of Orthogonal Tilted Fiber Bragg Gratings. Sci. Rep..

[14] Zheng D., Madrigal J., Chen H.L., Barrera D., Sales S. (2017). Multicore fiber-Bragg-grating-based directional curvature sensor interrogated by a broadband source with a sinusoidal spectrum. Opt. Lett..

[15] Barrera D., Madrigal J., Sales S. (2018). Long Period Gratings in Multicore Optical Fibers for Directional Curvature Sensor Implementation. J. Lightw. Technol..

[16] Wang S., Zhang W.G., Chen L., Zhang Y.X., Geng P.C., Zhang Y.S., Yan T.Y., Yu L., Hu W., Li Y.P. (2017). Two-dimensional microbend sensor based on long-period fiber gratings in an isosceles triangle arrangement three-core fiber. Opt. Lett..

[17] Tian Y., Chai Q., Tan T., Mu B.X., Liu Q., Liu Y.L., Ren J., Zhang J.Z., Oh K., Lewis E. (2018). Directional Bending Sensor Based on a Dual Side-Hole Fiber Mach–Zehnder Interferometer. IEEE Photon. Technol. Lett..

[18] Kong Y.J., Shu X.W. (2021). In-Fiber Hybrid Cladding Waveguide by Femtosecond Inscription for Two-Dimensional Vector Bend Sensing. J. Lightw. Technol..

[19] Ding L., Li Y., Zhou C., Hu M., Xiong Y.L., Zeng Z.L. (2019). In-Fiber Mach-Zehnder Interferometer Based on Three-Core Fiber for Measurement of Directional Bending. Sensors.

[20] Yang J., Guan C.Y., Zhang J.M., Wang M.J., Yang M., Zhu Z., Wang P.F., Yang J., Yuan L.B. (2019). Low-temperature crosstalk and surrounding refractive index insensitive vector bending sensor based on hole-assistant dual-core fiber. Appl. Opt..

[21] Yang J., Zou F., Guan C.Y., Ye P., Gao S., Zhu Z., Li P., Shi J.H., Yang J., Yuan L.B. (2022). Two-dimensional vector bending sensor based on a hole-assisted three-core fiber coupler. Opt. Lett..

[22] Wang L., Zhang Y.X., Zhang W.G., Kong L.X., Li Z., Chen G.T., Yang J., Kang X.X., Yan T.Y. (2019). Two-dimensional microbend sensor based on the 2-core fiber with hump-shaped taper fiber structure. Opt. Fiber Technol..

[23] Amorebieta J., Ortega-Gomez A., Durana G., Fernández R., Antonio-Lopez E., Schülzgen A., Zubia J., Amezcua-Correa R., Villatoro J. (2021). Compact omnidirectional multicore fiber-based vector bending sensor. Sci. Rep..

[24] Villatoro J., Amorebieta J., Ortega-Gomez A., Antonio-Lopez E., Zubia J., Schülzgen A., Amezcua-Correa R. (2020). Composed multicore fiber structure for direction-sensitive curvature monitoring. APL Photonics.

[25] Zhang S.X., Liu Y., Guo H.Y., Zhou A., Yuan L.B. (2019). Highly Sensitive Vector Curvature Sensor Based on Two Juxtaposed Fiber Michelson Interferometers with Vernier-Like Effect. IEEE Sens. J..

[26] Blanchard P.M., Burnett J.G., Erry G.R.G., Greenaway A.H., Harrison P., Mangan B., Knight J.C., Russell P.S., Gander M.J., McBride R. (2000). Two-dimensional bend sensing with a single, multi-core optical fiber. Smart Mater. Struct..

[27] Ouyang Y., Guo H.Y., Ouyang X.W., Zhao Y.J., Zheng Z., Zhou C.M., Zhou A. (2017). An In-Fiber Dual Air-Cavity Fabry–Perot Interferometer for Simultaneous Measurement of Strain and Directional Bend. IEEE Sens. J..

[28] Qi B.B., Su B.J., Zhang F., Xu O., Qin Y.W. (2022). Temperature-Insensitive Two-Dimensional Vector Bending Sensor Based on Fabry-Pérot Interferometer Incorporating a Seven-Core Fiber. IEEE Photon. J..

[29] Oliveira R., Cardoso M., Rocha A.M. (2022). Two-dimensional vector bending sensor based on Fabry-Pérot cavities in a multicore fiber. Opt. Express.

[30] Xiong C., Wang C.Y., Yu R.W., Ji W., Qin Y., Shen Y.C., Chen W., Liu A.Q., Xiao L.M. (2024). 3D printed multicore fiber-tip discriminative sensor for magnetic field and temperature measurements. Light Adv. Manuf..

[31] Oliveira R., Bilro L., Nogueira R. (2018). Fabry-Pérot cavities based on photopolymerizable resins for sensing applications. Opt. Mater. Express.

[32] Yang A.A., Bao W.J., Chen F.Y., Li X.Y., Wang R.H., Wang Y.P., Qiao X.G. (2023). Two-dimensional displacement (bending) sensor based on cascaded Fabry–Perot interferometers fabricated in a seven-core fiber. Opt. Express.

[33] Zhang C., Fu S.N., Tang M., Liu D.M. (2020). Parallel Fabry-Perot interferometers fabricated on multicore-fiber for temperature and strain discriminative sensing. Opt. Express.

[34] Wei G.Z., Jiang Q. (2020). Force sensitivity and fringe contrast characteristics of spheroidal Fabry-Perot interferometers. Opt. Express.

[35] Kong L.X., Zhang Y.X., Zhang W.G., Zhang Y.S., Yu L., Wang S., Geng P.C., Yan T.Y. (2018). High-sensitivity and fast-response fiber-optic micro-thermometer based on a piano-concave Fabry-Perot cavity filled with PDMS. Sens. Actuators A-Phys..

[36] Cui J.X., Luo H.J., Lu J.N., Cheng X., Tam H.Y. (2021). Random forest assisted vector displacement sensor based on a multicore fiber. Opt. Express.

[37] Liao C.R., Hu T.Y., Wang D.N. (2012). Optical fiber Fabry-Perot interferometer cavity fabricated by femtosecond laser micromachining and fusion splicing for refractive index sensing. Opt. Express.

[38] Cibula E., Donlagic D. (2010). Low-loss semi-reflective in-fiber mirrors. Opt. Express.

[39] Ji W., Shen Z.H., Yu R.W., Wang C.Y., Yang Z.Y., Liu L.Y., Xu L., Chen W., Chiang K.S., Xiao L.M. (2022). Spacing-Tailored Multicore Fiber Interface for Efficient FIFO Devices. J. Lightw. Technol..

[40] Zhou P., Zhang W.B., Wang J.X., Liu J., Su R.X., Wang X.M. (2016). Multimode optical fiber surface plasmon resonance signal processing based on the Fourier Series Fitting. Plasmonics.

